# Promoting Pollinating Insects in Intensive Agricultural Matrices: Field-Scale Experimental Manipulation of Hay-Meadow Mowing Regimes and Its Effects on Bees

**DOI:** 10.1371/journal.pone.0085635

**Published:** 2014-01-09

**Authors:** Pierrick Buri, Jean-Yves Humbert, Raphaël Arlettaz

**Affiliations:** 1 Division of Conservation Biology, Institute of Ecology and Evolution, University of Bern, Bern, Switzerland; 2 Swiss Ornithological Institute, Valais Field Station, Sion, Switzerland; Universidade de São Paulo, Faculdade de Filosofia Ciências e Letras de Ribeirão Preto, Brazil

## Abstract

Bees are a key component of biodiversity as they ensure a crucial ecosystem service: pollination. This ecosystem service is nowadays threatened, because bees suffer from agricultural intensification. Yet, bees rarely benefit from the measures established to promote biodiversity in farmland, such as agri-environment schemes (AES). We experimentally tested if the spatio-temporal modification of mowing regimes within extensively managed hay meadows, a widespread AES, can promote bees. We applied a randomized block design, replicated 12 times across the Swiss lowlands, that consisted of three different mowing treatments: 1) first cut not before 15 June (conventional regime for meadows within Swiss AES); 2) first cut not before 15 June, as treatment 1 but with 15% of area left uncut serving as a refuge; 3) first cut not before 15 July. Bees were collected with pan traps, twice during the vegetation season (before and after mowing). Wild bee abundance and species richness significantly increased in meadows where uncut refuges were left, in comparison to meadows without refuges: there was both an immediate (within year) and cumulative (from one year to the following) positive effect of the uncut refuge treatment. An immediate positive effect of delayed mowing was also evidenced in both wild bees and honey bees. Conventional AES could easily accommodate such a simple management prescription that promotes farmland biodiversity and is likely to enhance pollination services.

## Introduction

Animal pollination is an essential ecosystem service, without which more than 80% of flowering plants could not properly set seeds [1] and many important food products would become difficult to grow [2]. Despite its key role, pollination is nowadays threatened by numerous factors [3]. For example, managed honey bees *Apis mellifera* are impacted by a global colony loss, which has recently decimated up to 53% of European colonies [3], [4]. Alternative pollinators that are not directly managed by humans, like wild bees, are also threatened by habitat loss, landscape fragmentation, use of agro-chemicals, and general degradation of ecological resources [5], [6]. This phenomenon is referred to as the “pollination crisis” [7] (but see [8]). The pollination role of wild bees in food production has long been debated, but recent studies indicate it might be much more important than previously thought [9], [10]. The situation for pollinators is likely to worsen in the future due to the rising demand for food production that will inevitably lead to further agricultural intensification, which will in turn translate into even more demand for, and pressure on pollination [10], [11].

The intensification of farming practices has been the main process eroding biodiversity in low-input farmland, which was the typical cultivated landscape across Europe until World War II [12]–[14]. Intensification is achieved via the application of fertilizers and pesticides, and through a growing reliance on heavy machinery that necessitates radical landscape simplification [15]. In order to counter this negative impact of agricultural intensification on biodiversity, agri-environment schemes (AES) were established in the early 1990’s throughout Europe in order to encourage farmers to adopt more environmental friendly farming practices [16]. AES primarily aim at protecting and restoring farmland biodiversity, thus securing or even enhancing several ecosystem services, including pollination. Some AES like the sown wildflower strips and areas were specifically designed to promote pollinating insects, but their temporary based management misses the restoration of semi-natural habitats [17]. In comparison, extensively managed (low-input) grasslands established under AES regulations are widespread [16], usually harbouring more wild bees than high-intensity grasslands [18], [19]. However, several studies have established that these AES have only a moderate positive impact on overall biodiversity and bees [20]. This calls for the development of more appropriate management practices that can favour biodiversity, including pollinating insects [21].

The main aim of this study was to test whether and how slight modifications of mowing regimes may improve wild bee biodiversity in extensively managed hay meadows, a widespread AES scheme [22]. These altered management regimes had to be easily implementable by farmers to ensure their potential future uptake; they consisted of 1) not mowing a fraction of the meadow so as to leave an uncut area as refuge that is expected to boost wild bee biodiversity by continuously providing them with food resources, essentially nectar and pollen, during the entire season; 2) delaying by one month the first cut in order to provide undisturbed meadows with flowers and other crucial resources during the whole peak of natural wild bee activity. These two experimental treatments were compared with the Swiss AES serving as ‘control’; the ecological compensation areas (ECA). Standard management for hay meadows complying with ECA regulations are: first cut not before 15 June and; with no uncut refuge left behind. These treatments were applied at the field scale, two years in a row (2010 and 2011), in order to test for both immediate (within season) and cumulative (from one year to the following) effects. For this purpose, wild bees were collected twice in 2011: firstly in June, before the onset of any mowing intervention in all meadows; secondly beginning of July, when meadows with uncut refuges and control meadows were mown, but not meadows with delayed mowing regimes. To our knowledge, this study is the first attempt to manipulate mowing regimes at the field scale to test whether such simple measures can promote bee diversity. Although drawn from the Swiss context, the resulting recommendations have far-reaching implications for the establishment of AES across Europe if not beyond. They will contribute to the development and implementation of pollinator-friendly management practices and could potentially complement wild-flower strips [23]. They are also timely given the current intention of the European Union to frame a more biodiversity-friendly common agricultural policy [23]. In order to avoid jeopardizing essential components of biodiversity such as the guilds of natural pollinators, innovative farming practices have to be developed. We also take the opportunity to investigate and provide new data about the effectiveness our sampling method.

## Materials and Methods

### Ethic Statement

Farmers that participated to this project were informed about, and approved, the studies before they started. No endangered or protected species were sampled in this study.

### Study Sites

We selected 36 extensively managed hay meadows registered as ECA across the Swiss Plateau (lowlands between the Jura and the Alps) in 2010 (see Appendix S1 and S2). The Swiss Plateau can be characterized mainly as a simple landscape where non-crop habitats are still present, but constitute usually only 1–20% of the matrix [14]. The ECA retained for our experiments had to be registered since latest 2004 (range: 1993–2004) and had to have a minimal area of 0.3 ha (range: 0.3–1.7 ha). The meadows were situated between 390 and 833 m altitude. They were clustered in 12 study areas (our geographic replicates) distant from each other by ≥5 km, each area containing three meadows that were more than 400 m distant (range: 440–6170 m) but that were enclosed within a radius of 3.5 km.

We had first to assess the different land covers as these could be important covariates that should be accounted for in our analysis. Land covers were extracted from the Vector 25 data base of the Swiss Federal Office of Topography [24], using QGIS [25] and SpatiaLite [26] software. Land covers [proportion of forest, settlements, water bodies (including rivers), special crops (vineyards and orchards), and gravel pits] were quantified around each meadow within different nested concentric radii ranging from 250 to 3000 m, with steps of 250 m. A principal component analysis (PCA) was then conducted on land covers to draw synthetic information about the various landscape contexts at the different geographic replicates [27], [28]. The PCA was performed with land cover values averaged across these radii, this to avoid auto-correlation [28]–[30]. We retained only PCA axes that had a proportion of variance superior to a broken-stick model with heuristic for principal component selection [31], with the function *PCAsignificance* of the package BiodiversityR [32]. Then the coefficients of the Pearson product-moment correlation (eigenvectors) of the retained axes were used to select important land covers with 0.5 as cutting of value.

### Study Design

A randomized block design was adopted [33], where the three mowing regimes (our two experimental treatments and a control) were applied once each within each study area. Hence, each area represented a geographic replicate (*n* = 12), i.e. an experimental block in the design, which allowed achieving data independency. The following treatments and control were randomly assigned to the three meadows within each area. We start with the control, because it represents the standard management that today prevails among extensively managed hay meadows within the ECA (ecological compensation areas) measures of the Swiss AES: 1) control meadow (abbreviated C, C-meadow): managed according to the Swiss regulations for ECA extensive hay meadows, i.e. first cut not before 15 June; 2) refuge treatment (R, R-meadow): same as C, but at each cut 10 to 20% of the meadow area were left uncut; 3) delayed mowing (D, D-meadow): same as C, but first possible cut not before 15 July (one month later than C). All other management aspects (such as non-application of fertilizers and pesticides or minimal duration of 6 years) abided by the present regulation [34]. Each farmer was interviewed about mowing dates and related management issues using a standardized questionnaire.

### Wild Bees Sampling

In 2011, plastic bowl traps (13 cm in diameter and 12.5 cm deep) were used to sample wild bees (Hymenoptera: Apoidea) applying the following protocol: three bowl traps (blue, white and yellow) were fixed on a wooden pole just above the grass vegetation layer [35]. They were operated at daylight (8∶00–19∶00) during one day in order to avoid local population depletion [36]. Since this study aimed at comparing relative differences among mowing regimes and not at providing a full inventory *per se*, such standardized operating time was considered as being sufficient [37]–[41]. Three such poles equipped with three bowl traps were placed at the apexes of a virtual isosceles triangle (base: 14 m; sides: 10 m) randomly placed inside the meadow, within at least 10 m from meadow edges so as to reduce margin effects [18]. Meadows were sampled twice, a first time between 23 May and 14 June, i.e. before the onset of mowing in any treatment and control meadows (hereafter referred to as the ‘June’ samples) and a second time between 2 July and 12 July, i.e. before the first mowing of D-meadows but when C-meadows and R-meadows were regrowing (hereafter ‘July’ samples). Samplings took place on sunny, non-windy days with ambient temperature ≥15°C [35]. All the meadows within a given area were sampled simultaneously (Appendix S2 for exact dates). Samples were stored individually in plastic bags and frozen at −20°C. Before sorting them, defrozen samples were washed; bees pinned and dried [37]. Bees were identified according to identification keys for Central Europe [42]–[48].

### Data Analysis

Data were analysed with generalized linear mixed models (GLMMs) using the *lmer* function from the *lme4* package for R [49]. Wild bees consisted of the so-called “solitary bees” and of bumblebees pooled together. Fixed effects were the mowing treatments and the land covers selected in the previous part. The latter were added in the models, progressively increasing model complexity, following a bidirectional stepwise procedure [50], [51]. Areas (our geographic replicates) were designated as a random effect. Response variables were pooled for each meadow and resulted in: wild bee abundance; species richness and; diversity (Shannon-Wiener index), the former two variables were analysed fitting a Poisson error distribution and the latter one fitted a normal error distribution. Data of the two sampling periods (June and July) were first analysed pooled together, then separately, this in order to better appraise underlying patterns. Planned orthogonal comparisons were done to identify significant differences between the treatments. In addition, we also investigated in a similar way the effects of these mowing regimes on the abundance of managed honey bees, given that feral honey bee colonies apparently do not occur in Switzerland [52] and the effectiveness of the different colours of our traps. All the analyses were performed with statistical software R version 2.15.0 [53].

## Results

We collected a total of 1′620 wild bees (Appendix S2) and 281 honey bees. Cryptic, sibling species of bumblebees that were difficult to identify were grouped within their respective taxonomic groups, mostly subgenera (*Bombus* sensu stricto, *Megabombus* and *Thoracobombus* [47]). Cryptic, sibling species of solitary bees were grouped within the following categories: *Halictus simplex* group (*Halictus simplex*; *H. eurygnathus* and *H. langobardicus* and *Andrena ovatula* group *(Andrena ovatula* and *A. albofasciata*). Altogether, we could identify 62 wild bee species (9 bumblebee and 56 solitary bee species; full species list in Appendix S3).

### Bowl Trap Efficiency

Yellow bowl traps were generally more efficient (greater number of captures of wild bees) than white traps which were themselves more attractive than the blue ones. These differences were significant when the June and July samples were pooled, and when the June data was considered separately. In July, however, yellow and white traps did not differ in efficiency between each other though they were still more attractive than the blue traps (detailed analysis in Appendix S4).

### Management and Land Cover

Our study meadows were mown, on average (±SD), 1.92 times (0.56) and 1.81 times (0.49) in 2010 and 2011, respectively, with a minimum number of cuts of one and a maximum of three. There was no significant difference in the yearly number of cuts between 2010 and 2011. In 2011, the first cut took place between 15 June and 26 June in C– and R–meadows, and between 15 July and 15 August in D–meadows (exact dates are provided in the appendix S5). In R-meadows, uncut grass refuges covered, on average, 15% of the meadow area.

Regarding the PCA on landscape covers, only the first component fulfilled the broken-stick criteria (73.41% of variance explained vs 45.66% expected). The following land covers were identified as significant based on their eigenvalues (Pearson product-moment correlation) and retained for subsequent analyses: forest (−0.511); special crops (0.566) and water bodies (0.515).

### Effect of Mowing Treatments on Wild Bees

**Figure 1:**
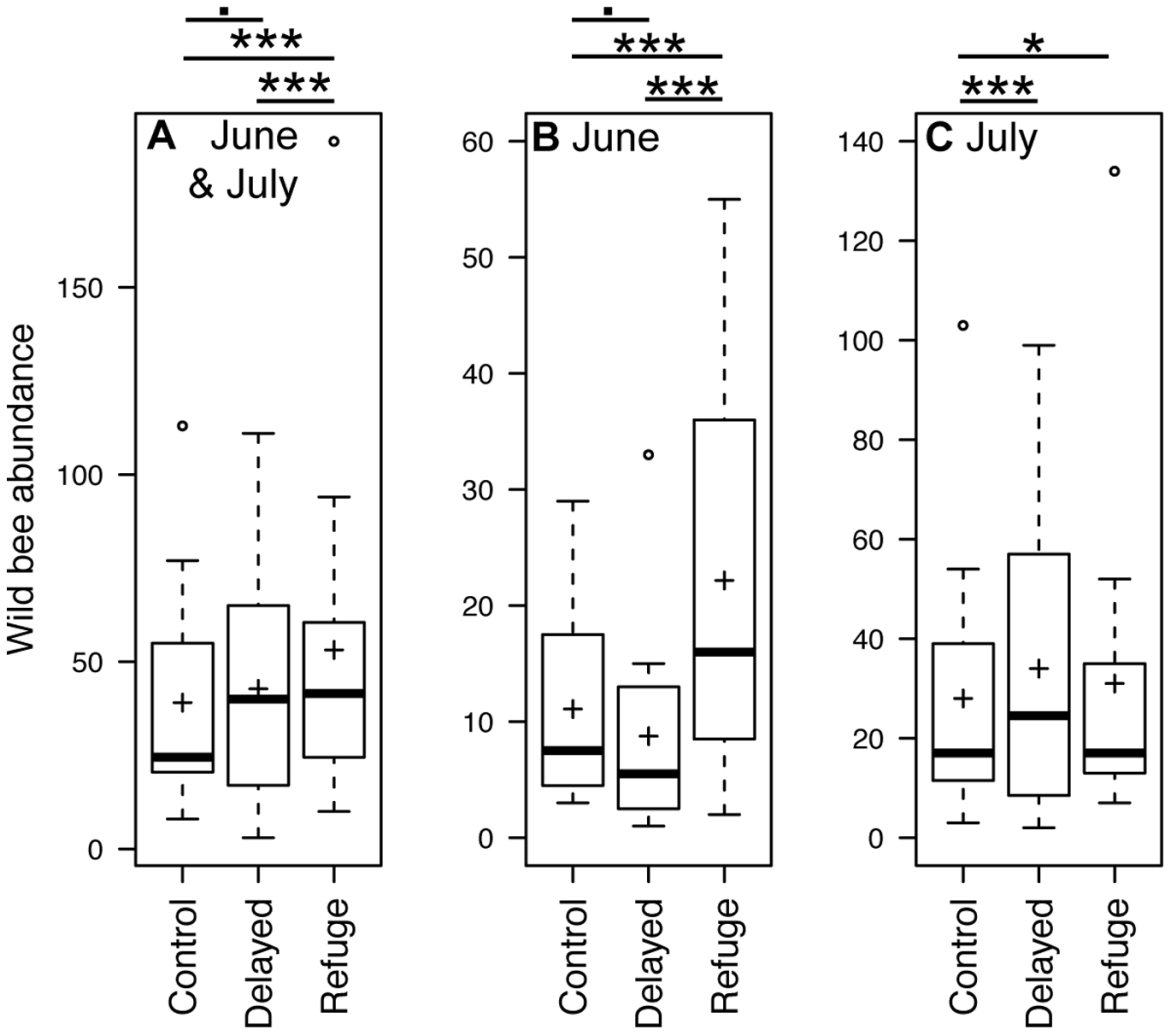
Abundance of wild bees. Number of individuals captured according to the different mowing treatments in: a) June and July (pooled data); b) June only; and c) July only. Bold transversal bars represent medians;+the means; box boundaries the first and last quartiles; whiskers the inter-quartile distance multiplied by 1.5; and open dots the outliers. Significance codes of statistical tests: · marginally significant results (0.1<*P*<0.05); *significant results, *P*<0.05; ***highly significant results, *P*<0.001.

**Figure 2:**
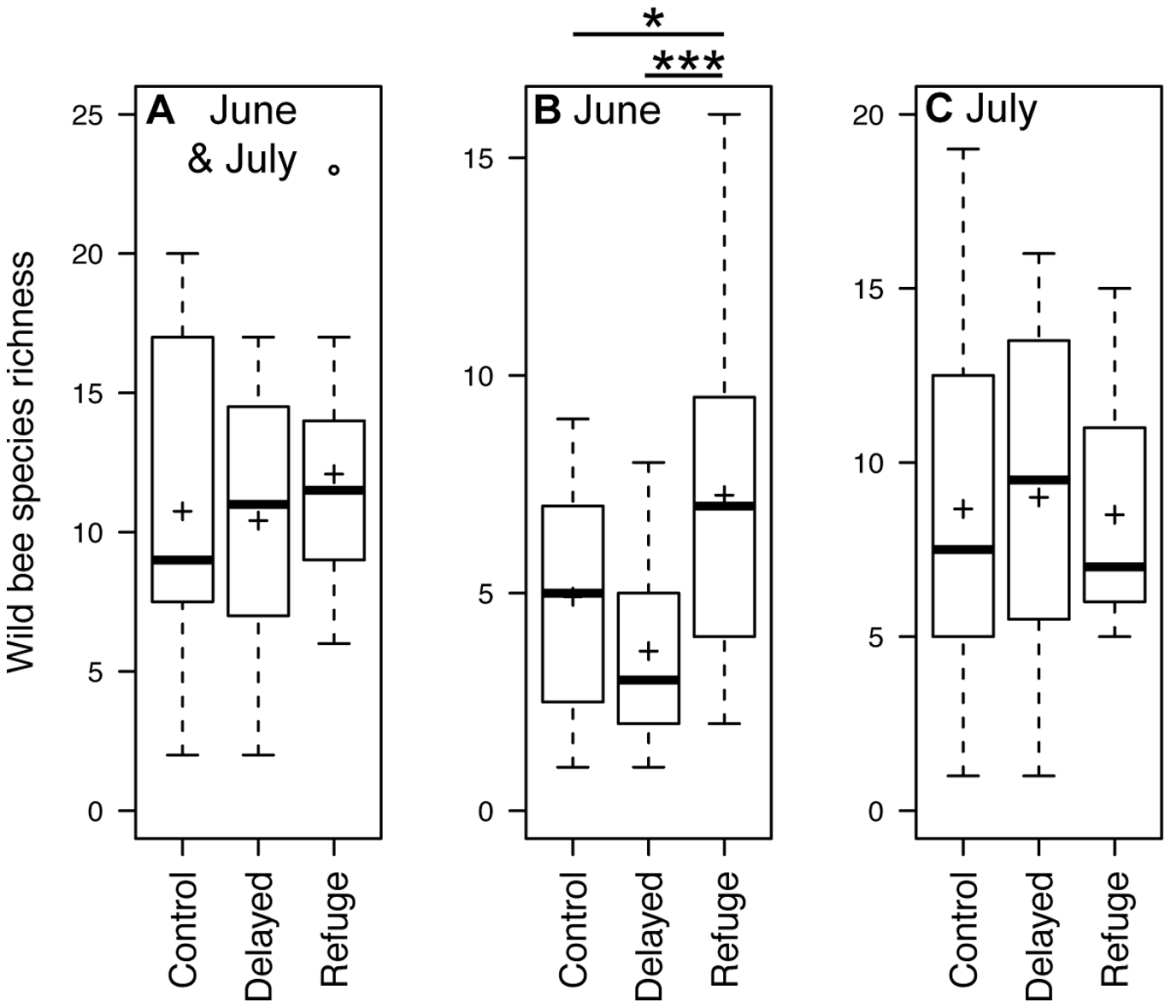
Species richness of wild bees. Number of species captured according to the different mowing treatments in: a) June and July (pooled data); b) June only; and c) July only. Symbols as in Fig. 1.

In the analyses performed with data from June and July pooled together, the mean abundance (±SE) of wild bees was 53.16 (±14.15) in R-meadows (refuge) and was significantly higher compared to C-meadows (control, 39.08±8.9; Fig. 1a and Table 1). Abundance in D-meadows (delayed mowing) was only marginally higher than in C-meadows. Finally, significantly fewer individuals were found in D-meadows compared to R-meadows (*Z = *3.677, *P*<0.001). The land covers retained in this first model were forest and water bodies with both a negative effect on wild bee abundance; in contrast, special crops had a positive effect. Species richness did not show any significant difference among the mowing regimes with the June and July samples pooled (Fig. 2a and Table 2). Neither did we find any difference for the Shannon-Wiener index of diversity for pooled data.

**Table 1:** Abundance of wild bees.

	Total		June		July	
Parameters	*Z*-value	*P*-value	*Z*-value	*P*-value	*Z*-value	*P*-value
Delayed	1.713	0.086	−1.927	0.054	3.594	**<0.001**
Refuge	4.036	**<0.001**	5.487	**<0.001**	2.472	**0. 0.013**
Forest	−2.843	**0.005**	–	–	−2.319	**0. 021**
Water bodies	−2.683	**0.00730**	–	–	−4.021	**<0.001**
Special crops	2.669	**0.008**	2.044	**0.041**	–	–

GLMM outputs on the abundance of the wild bees recorded according to the different managements and the most important land covers. Analyses presented are the pooled data (June and July added); the June sampling session and; the sampling of July. Significant contrasts are highlighted in bold.

**Table 2:** Species richness of wild bees.

	Total		June		July	
Parameters	*Z*-value	*P*-value	*Z*-value	*P*-value	*Z*-value	*P*-value
Delayed	−0.251	0.802	1.490	0.1363	0.274	0.784
Refuge	0.965	0.335	2.044	**0.036**	−0.139	0.889
Forest	–	–	–	–	–	–
Water bodies	–	–	–	–	–	–
Special crops	–	–	2.044	0.041	–	–

GLMM outputs on the species richness of the wild bees recorded according to the different managements and the most important land covers. Analyses are presented in the same way as Table 1.

In June, the abundance of wild bees in R-meadows was, on average, 22.17 (±5.05), i.e. significantly higher than in C-meadows (11.08±2.44) and D-meadows (8.75±2.60; *Z = *2.101, *P* = 0.035; Fig. 1b). Abundance in D-meadows was also marginally lower than in C-meadows. Only special crops were retained as a significant land cover in this model, with a positive effect. Species richness in R-meadows was 7.25 (±1.15), significantly higher than in both C-meadows (4.92±0.80; Table 2) and D-meadows (3.67±0.58; Z = 3.664, *P*<0.001; Fig. 2b). Special crops coverage had again a positive effect on species richness. In contrast, we detected no significant effect on the Shannon-Wiener index of diversity.

In July, the mean abundance of wild bees collected in D-meadows was 34.00 (±8.78) individuals, i.e. significantly higher than in C-meadows (28.00±8.17; Fig. 1c). The abundance in R-meadows (31±10.07) was also higher than in C-meadows (*Z = *2.472, *P = *0. 0013). Forest (*Z = *2.319, *P = *0. 021) and water bodies (*Z* = −4.021, *P*<0.001) were the only land covers retained by the model; both had a significant negative effect. Neither species richness nor the Shannon-Wiener index of diversity showed any significant difference among mowing regimes.

When the data of June and July were pooled, honey bee abundance was, on average, 9.91±2.49 in D-meadows, i.e. significantly higher than the abundance recorded in C-meadows (6.5±1.08; GLMM with Poisson; *Z = *2.894, *P = *0.0038) and R-meadows (6.83±1.38, Z = 0.316, *P* = 0.75). In June, no significant difference was found, while in July honey bee abundance was significantly higher in D-meadows (6.83±2.05) compared with C-meadows (3.67±0.88; *Z = *3.323, *P*<0.001) and R-meadows (3.08±0.91; *Z = *3.221, *P = *0.00128).

## Discussion

This study shows that leaving 10–20% of the area of an extensive meadow uncut when mowing (R-meadows) is overall beneficial for wild bee populations, more so than delaying the date of mowing by one month (D-meadows). There were variations in the observed pattern according to whether we consider immediate (within the same season) or cumulative (from one year to the following) effects. Regarding cumulative effects [samples collected in June in the year following (i.e. year_t+1_) the year of first application (year_t_) of the different management treatments, but before any mowing event in year_t+1_], positive effects were evidenced for both wild bee abundance and species richness. The average wild bee abundance was double so high in R-meadows compared to C-meadows, and even 2.4 times higher than in C-meadows that had the lowest values (Fig. 1b). Species richness was, on average, 1.75 and 1.4 higher in R-meadows compared to D-meadows and C-meadows, respectively (Fig. 2b). Immediate effects showed a reversed pattern, but only regarding wild bee abundance, with D-meadows harbouring, on average, 1.2 and 1.1 times more individuals than C- meadows and R-meadows, respectively; this is not very surprising given that D-meadows were not yet mown at the second sampling session. Concerning, honey bee population size estimates, we could not evidence a cumulative effect, while immediate effects showed that D-meadows supported ca. 1.8 and 2.3 times greater abundances than C-meadows and R-meadows, respectively.

The positive cumulative effect of the refuge treatment (R) on wild bee abundance indicates that populations could build up thanks to the grass refuge installed the year before. This demonstrates that uncut grass refuges have a positive impact on these pollinating insects beyond the season they are applied in. This cumulative effect is crucial for the maintenance of pollination services because pollination efficiency is based on the redundancy principle, which emphasizes the importance of pollinator abundance above species richness [7], [54], [55]. Our results further confirm that wild bees can react extremely rapidly to changes in management practices: this first analysis stems from just one year of field experimentation (June 2010– July 2011). Such a rapid positive reaction is consistent with the responses of bumblebees to modifications in grazing management [56] and manipulation of the cutting management of flower patches [27]. Similar responses were also observed in other taxa, such as orthopterans [57], spiders [58] and the only other pollinator taxon studied, butterflies [59]. Finally, the absence of a similar effect in honey bees in the present study can be due to the fact that these Hymenoptera depend neither on the structures nor on the food resources offered by the refuges for building their colonies, while they furthermore profit from artificial feeding at the hives (feral honey bees are extremely rare in Switzerland [52]). The continuation of our experiments during the coming years will allow assessing whether cumulative effects may further grow with additional years of implementation of the treatments.

Regarding immediate effects, delayed mowing (D-meadows) appeared to be more efficient than the creation of an uncut refuge (R-meadows) for increasing abundance of both wild and honey bees. Yet, the magnitude of these positive effects was strikingly lower than the cumulative effects obtained with the refuge treatment. Furthermore, this effect reflects the fact that D-meadows were not yet mown at the time of the second sampling session, contrary to R-meadows and C-meadows that were already regrowing after the first cut. These D-meadows were thus the main sources of nectar and pollen left in the agricultural matrix at that time of the year, which corresponds to the peak of hymenopteran pollinator activity [27], thus typically generating some short-term spatio-temporal concentration [60]. This hypothesis of a temporary concentration effect is further supported by the lower wild bee abundance and species richness in D-meadows compared to C- and R-meadows in June: for many species that firstly depend on vegetation with a late phenology, mowing around mid-July could still be too early. Notwithstanding the fact that bowl trapping is not the most efficient method to capture honey bees [35], [61], their concentration in D-meadows in July highlights the need for valuable flowering patches at this time of the year. Maintaining uncut meadows in the middle of the summer could indeed provide them with precious floral resources between the massive spring blossoming of both natural flowers or some crops (mainly oilseed rape *Brassica napus*, L.) and other crops with a later phenology, e.g. sunflowers *Helianthus annuus* Linnaeus, 1753 [62].

Improvement of species richness was only detected as a cumulative effect (June samples) and occurred furthermore only in R-meadows, but not in D-meadows. This result is in accordance with the outcome of the main study on Hymenoptera retrieved in the meta-analysis on delaying mowing done by Humbert et al. [63]. To the contrary of the main trend for arthropods, no effect of postponing mowing could be evidenced for bumblebee species richness [64].

Surprisingly, the effect of our mowing treatments did not affect species diversity (Shannon-Wiener index). An explanation could be that the relative population sizes of different sympatric wild bee species do not vary in relation to the number of co-occurring species [65], which would lead to little spatial variation in the index. Moreover, although R-meadows harboured, on average, more species than C-meadows and D-meadows in June, there was no new species specifically profiting from the refuge that appeared in the samples. Actually, among the 62 different species captured, only twelve occurred in more than seven of the 12 study areas (Appendix S4). This high level of spatial differentiation in bee communities, i.e. apparent high level of functional redundancy, was particularly striking within bee genera having similar ecological requirements, such as *Lasioglossum* and *Halictus* [6].

Land covers have an important influence on bees that are relatively mobile organisms [28]–[30]. The major land covers selected through the PCA were forest, water bodies and special land managements. The two first ones had the most part of time a negative influence, because they represent less suitable habitats for bees. Thus a high proportion of such features in the surroundings have a negative influence. Special land management had a positive influence in spring. This could be due to the kind of crops present in this land cover, especially orchards that are reputed to be major nectar sources.

Concerning the difference observed between the colours of the traps, the conclusion of the effectiveness of the yellow is in accordance with the literature [35]. Interestingly, other colours, especially white, can be as effective and more representative of the plant flowering community and thus illustrates the complementarity of the different colours for this traps.

## Management Recommendations and Conclusions

This study constitutes to our knowledge the first attempt to experimentally test, moreover at real field-scale, the effects of different grassland management regimes in hay meadowland on wild bee communities and population sizes. It demonstrates that creating uncut refuges on a relatively small fraction of a hay meadow can quickly and efficiently promote pollinating insects such as wild bees during the following year, which is likely to enhance an essential ecosystem service. Although it remains to be established whether the inter-annual positive effects we observed further cumulate beyond one year, this measure represents a promising agri-environmental option, especially given that its simplicity of implementation might ensure a quick up-take by farmers, of course providing that financial incentives exist. In contrast, delaying mowing seems to have comparatively much smaller positive effects on bees as it simply leads to a temporary concentration of bees on the few patches with flowering plants that remain in farmland matrices that otherwise become hostile for pollinators after late spring mowing operations. Uncut refuges could enter the toolkit for promoting pollinators within farmland, similar to, for instance, wildflower sown margins [27].

Another advantage of the uncut refuge option, over the delayed mowing option, is that it does not affect hay production to the same extent, given that only a fraction of the meadow remains unmown. The hay extracted from the non-refuge area would furthermore be of overall better quality because the timing of mowing operations can take place earlier than in D-meadows, i.e. closer to the period of forage quality peak. A systematic implementation of this measure within extensive hay meadows across the agricultural matrix might efficiently boost wild bee populations and communities. We may furthermore expect that the overall impact of a network of such refugial structures reaches beyond the sum of the local effects, due to opportunities for reconstituting functional meta-populations and integral communities, this especially given the short flight radius of numerous pollinators [29], [30]. This simple measure could also easily be integrated in extant AES which – given the extension of grassland AES [20], [22] – would theoretically lead to widespread improvement of pollination services in agriculture. Finally, the fact that this measure is already suggested as a voluntary enrolment for farmers in such schemes will enhance the probability of its uptake [66], [67]. Future research must investigate whether extra positive cumulative effects will, in the mid and long run, add to the short-term effects observed in this study. It must also establish whether other plant and animal taxa benefit from uncut refuges, and whether combining this measure with delayed mowing on, for instance, another small fraction of the same meadow might multiply the benefits for biodiversity, especially pollinating insects.

## Supporting Information

Appendix S1 **Sampling sites.** Map of Switzerland with the lowland and mountainous areas as defined in the Swiss agricultural cadastre. Sampling points are indicated with coloured dots.
(PDF)

Appendix S2 **Geographic coordinates of all sampling sites with indication of total number of individuals trapped.** Numbers of individuals of solitary bees, bumblebees, honey bees and other insets caught are provided for each meadow.
(XLS)

Appendix S3 **List of the species of wild bees identified per area.** The nomenclature follows the one proposed in the identification keys of Félix Amiet.
(XLS)

Appendix S4 **Study of the effects of different colours of bowl traps.** Comprehensive analyses of the attractiveness of the different colours of the most numerous species.
(DOC)

Appendix S5 **List of the different mowing dates for the year 2011.** Dates were communicated by the farmers.
(XLSX)
