# Supplementary figures and images for: Promoting Pollinating Insects in Intensive Agricultural Matrices: Field-Scale Experimental Manipulation of Hay-Meadow Mowing Regimes and Its Effects on Bees
Source: PLoS One. 2014 Jan 9;9(1):e85635. doi: 10.1371/journal.pone.0085635 (PMC3887108; doi:10.1371/journal.pone.0085635)

Sampling points

- 15 July
- Control
- Refuge

Borders

Water bodies

Lowland

Mountainous areas

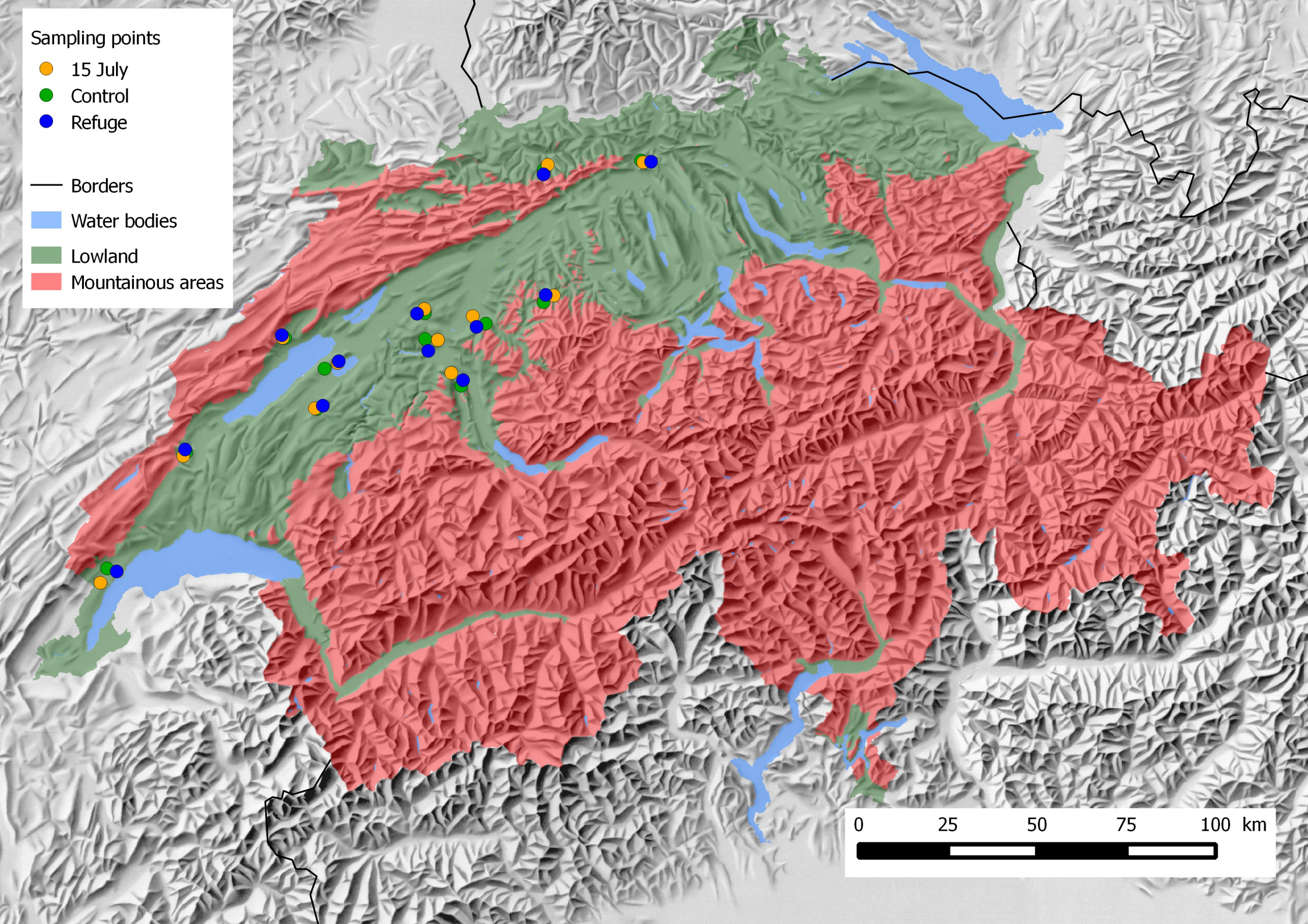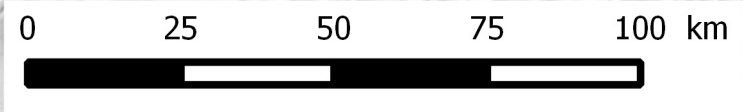

Supplement: Appendix S1 — Sampling sites. Map of Switzerland with the lowland and mountainous areas as defined in the Swiss agricultural cadastre. Sampling points are indicated with coloured dots. (PDF) [file pone.0085635.s001.pdf]
