# Supplementary material for: Promoting Pollinating Insects in Intensive Agricultural Matrices: Field-Scale Experimental Manipulation of Hay-Meadow Mowing Regimes and Its Effects on Bees
Source: PLoS One. 2014 Jan 9;9(1):e85635. doi: 10.1371/journal.pone.0085635 (PMC3887108; doi:10.1371/journal.pone.0085635)
Supplement: Appendix S4 — Study of the effects of different colours of bowl traps. Comprehensive analyses of the attractiveness of the different colours of the most numerous species. (DOC) [file pone.0085635.s004.doc]

**Supporting information S4: Study of the effects of different colours of bowl traps**

*Data analysis*

The attractiveness for wild bees of different colours of bowl traps were first analysed with standard GLMMs: abundance and species richness of captured wild bees served as response variables, bowl colours (yellow, white or blue) and experimental treatmentsas fixed effects, and sites as a random factor. Secondly, species-specific preferences were investigated using a model-based analysis of multivariate abundance, using the function *manyglm()* from the package *mvabund* (Wan*g et a*l. 2012). Multivariate abundance data analyses were performed only on species that were present in at least eight sampling areas so as to ensure sufficient statistical power and scientifically sound inferences. Data of the two sampling periods (June, July) were first pooled and then analysed separately.

*Results*

In the pooled analyses, the abundance observed (average ± SE) in yellow bowl traps was (20.58 ± 4.96) higher than in blue traps (8.42 ± 2.54; GLMM with Poisson; *Z* = -13.091, *P* <0.001) and white traps (16.00 ± 4.18; *Z* =-4.519, *P* < 0.001); white traps were in turn more efficient than blue traps (*Z* = 9.050, *P* < 0.001). In June, yellow traps collected, on average, a higher number (7.64 ± 2.46) than blue traps (2.41 ± 0.77; *Z* = -9.285, *P* <0.001) and white traps (3.95 ± 1.10; *Z* = -6.315, *P* < 0.001). In July, the abundances recorded in yellow (12.95 ± 3.68) and white (12.06 ± 3.75) traps were not different (*Z* = -1.103, *P* = 0.27), but blue traps (6.00 ± 2.01) caught significantly fewer wild bees than the two other colours; *Z* = -9.365, *P* < 0.001).

Species-specific preferences were then investigated in the more widespread species (present in ≥ 8 areas, out of 12). Twelve wild bee species met that criterion, with 8 showing significant differences regarding colour preference. Yellow traps caught more specimens than blue traps. White was in four species as successful as yellow (*Bombus sensus stricto* group, *Halictus tumulorum*, *Lasioglossum calceatum* and *Lasioglossum puaxillum*); it was even significantly more attractive than the other two colours in the *Halictus simplex* group (Table S3). Analyses carried out with separate seasonal datasets (June, July) showed the same patterns as with the pooled data; they are therefore not presented here.

### *Discussion*

Overall (June and July pooled), wild bees were mostly attracted by yellow traps, followed by white traps, and finally blue traps. In June, similar preferences were observed as with pooled seasonal samples. However, in July, no significant difference between yellow and white traps could be detected, though both were more efficient than the blue ones. This result shows that attractiveness of bowl traps can vary among species and according to season. It confirms the recommendations of Westphal et al. (2008) of using the three (yellow, blue and white) colours in parallel.

Despite this recommendation several studies have used only one or two colours (e.g. Tuell, Ascher, & Isaacs 2009; Krewenk*a et a*l. 2011). In our case, white traps were as successful in July as the yellow ones, probably because more flowers with white corolla were blooming at that time of the year (P. Buri, personal observations). Notwithstanding the fact that yellow remains the most attractive colour for measuring general bee abundance, this does not apply to all wild bee species, which calls for systematically using traps of different colorations. Four species were equally attracted by the yellow and white traps, while the *Halictus simplex* group was even more linked to white traps (Table S3). Regarding blue traps, they seemed quite unattractive in our experiment, although five individuals of four different species were caught only in blue traps (one *Bombus sylvestris*, one *Halictus confusus*, two *Chelostoma campanularum*, and one *Eucera longicornis*). We recognize that this pattern could emerge randomly, but cannot exclude the possibility that these species would have been missed if no blue traps were set.

**Table S2. Bowl traps colour attractiveness. Species or species groups included in the multivariate analysis, with indication of the total number of individuals trapped, the number of replicates that harboured the species or species group, bowl trap colour preference with respective *P*-values. For more details, see Fig. S3 that provides information about bee abundance vs bowl trap colours for each species or species group. Significant differences in bold.**

| **Species or species group** | **Number of individuals** | **Number of replicates** | **Bowl trap colour preference** | ***P*-value (adjusted)** |
| --- | --- | --- | --- | --- |
| *Halictus simplex group* | 177 | 10 | white > blue = yellow | **0.001** |
| *Halictus tumulorum* | 118 | 12 | yellow = white > blue | **0.001** |
| *Lasioglossum calceatum* | 144 | 11 | white > blue | **0.001** |
| *Lasioglossum malachurum* | 346 | 10 | yellow > white > blue | **0.001** |
| *Lasioglossum zonulum* | 181 | 12 | yellow > white > blue | **0.001** |
| *Lasioglossum pauxillum* | 91 | 10 | white > blue | **0.002** |
| *Bombus sensus stricto* | 89 | 12 | yellow > white = blue | **0.029** |
| *Halictus scabiosae* | 25 | 8 | yellow = white = blue | 0.111 |
| *Bombus lapidarius* | 28 | 8 | yellow = white = blue | 0.285 |
| *Lasioglossum leucozonium* | 21 | 8 | yellow = white = blue | 0.285 |
| *Bombus hortorum* | 18 | 8 | yellow = white = blue | 0.859 |
| *Bombus pascuorum* | 24 | 11 | yellow = white = blue | 0.859 |

**Figure S2. Mean (±SE)** number of wild bees trapped with respect to bowl trap colour. . Significance codes of statistical tests: ∙ marginally significant results (0.1 < *P* < 0.05); * significant results, *P* < 0.5;** very significant results, 0.01 <.*P* < 0.001; *** highly significant results, *P* < 0.001.


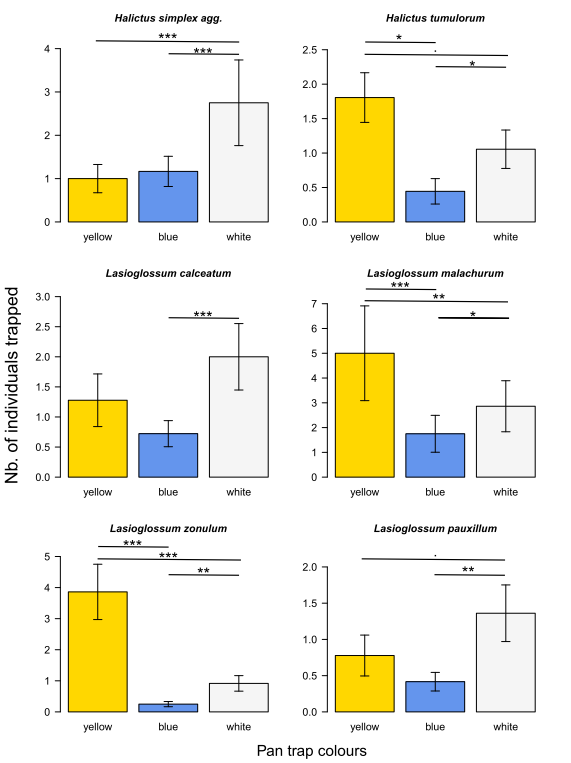


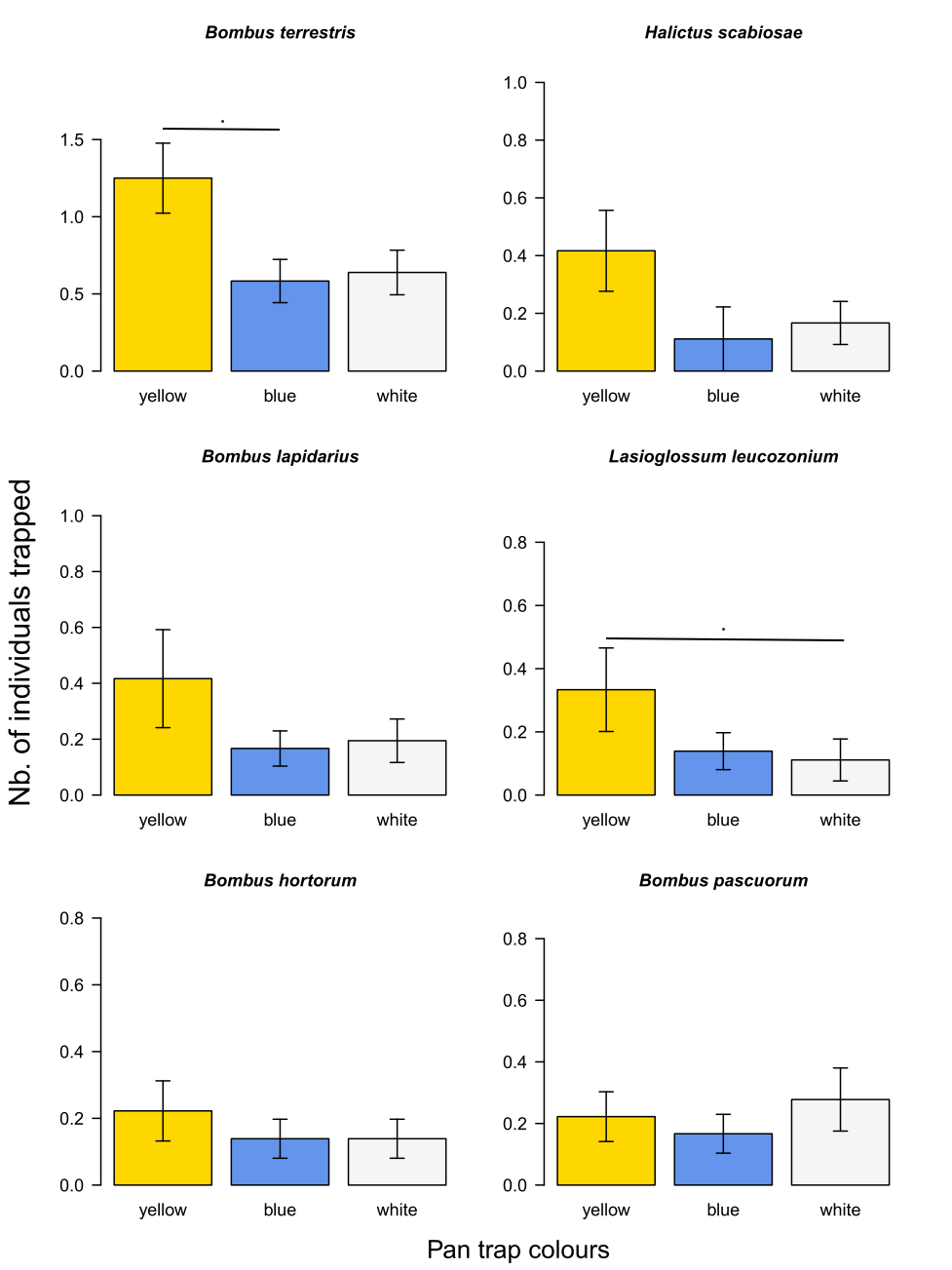


***References***

Krewenka, K.M., Holzschuh, A., Tscharntke, T. & Dormann, C.F. (2011) Landscape elements as potential barriers and corridors for bees, wasps and parasitoids. *Biological Conservation,* **144,** 1816-1825.

Tuell, J.K., Ascher, J.S. & Isaacs, R. (2009) Wild Bees (Hymenoptera : Apoidea : Anthophila) of the Michigan Highbush Blueberry Agroecosystem. *Conservation Biology and Biodiversity,* **102,** 275-287
